# Grand challenge: ELSI in a changing global environment

**DOI:** 10.3389/fgene.2013.00158

**Published:** 2013-08-16

**Authors:** Dov Greenbaum

**Affiliations:** Greenbaum Group, Department of Molecular Biophysics and Biochemistry, Yale UniversityLawrence, NY, USA

Contrary to conventions held by many scientists, academic research is not entirely conducted in a vacuum. And while it may be somewhat difficult for young and even established researchers to see the current or eventual impact of their often admittedly esoteric research, that impact still exists and should at a minimum be accounted for, if not fully examined.

In the United States, ethical and social concerns related to the results of basic science research were likely exacerbated out of a post-World War II fear of the awesome power and huge societal implications of what was otherwise thought of gentlemanly tinkering in the lab. With the rise of substantial government funding initially in physics and subsequently in biology, it was only a matter of time before Science acquiesced: acknowledging that they too should be interested in examining the ethical legal and social implications of their research.

While many appreciate the government's interventions in this area, others are quick to point out that a large standing army of academic ethicists, many woefully unfamiliar with actual industry practices outside the Ivory Tower, will inevitably find battles to find. Some of those battles may involve real and substantive issues. In other cases, ethical intervention may be an unfortunate, unnecessary, or overblown interference by academics looking to make their mark.

In some senses, the science of ethical, legal and social implications (ELSI) ameliorated this concern by establishing a governing set of goals and principles.

ELSI, in its modern incarnation, was born in a last minute revision of a 1988 James Watson speech, where Watson set aside funds specifically for the study of the ethical, legal, and social implications of the massive US government funded Human Genome Project (Cook-Deegan, 1994). This unilateral decision was notably the first act of Dr. Watson as the new director of the National Institutes of Health Human Genome Institute.

Of the massive outlay allotted to the Human Genome Project, a modest yet substantial three percent was set aside for the study of ELSI. The ELSI component has been a staple part of genomics grants ever since (Daniel Seltzer et al., 2011). In subsequent years of the project, between three to five percent was annually doled out to study the ELSI issues. As a result, in the past decades, hundreds of millions of dollars have been distributed under the auspices of the Human Genome Project ELSI program (National Advisory Council for Human Genome Research, 2008).

The 1990 ELSI Working group report set forward the research scope of ELSI Genomics (Working Group on Ethical, Legal, and Social Issues, 1990). The issues to be examined followed the description of the purpose of the parts of the program as per the first report by the ELSI Working Group. These goals included anticipating and addressing the implication for individuals and society for genomics, examining the ethical legal and social consequences of genomics, stimulating public discussion on the issues and developing policy options.

Notwithstanding the incredible strides forward over the past two decades in science and the technological applications within society, many of these original considerations are still relevant today, albeit in a broader scope.

ELSI has outgrown its modest beginnings and has become pertinent far beyond just the Genome Project. As it should.

In fact, ELSI matters today as much if not more so than at its inception. As scientists we are beholden to the tax payers that generously fund our research, and as a result we owe them, at minimum, the decency of acknowledging their funding by investigating how our research affects them. Other debts to the funders of science research include open access to the research, as exemplified by the Frontiers model.

However, that's not to say that research ought to be limited by the ethical social legal or even moral considerations of the public. This is particularly the case as the ethical, legal, and social implications of science are somewhat subjective, and can differ radically depending on time, place and jurisdiction. Thus, while the implications of research within society ought to be considered and those implications might be used to directly or indirectly guide science, they should rarely be used to limit science—this however is a non-trivial issue and should be addressed by ELSI.

What then ought the modern role of ELSI be?

- In general, the role of ELSI research should be to not only research, examine and report on the ethical legal and social implications of research, but it should also include, as an integral component, a true engagement of the lay public. This engagement can be in the form of lay publications, lay speaking, and/or the inclusion of lay professionals in the ELSI process (Henderson et al., 2012). ELSI should be more proactive in bridging the divide between science and other components of society, be it law, policy, other scientific fields or just the lay public in general. This is particularly important when one or more components of society encounters new and novel science of possible relevance (Walker and Morrisse, 2012).
- And ELSI needs to engage more rapidly. With the constant flux of science and society, what was important, relevant, or cutting edge last week might be out-of-date or obsolete this week. To this end, ELSI should take advantage of emerging tools such social media, microblogging and the like to push ELSI research forward and to further engage the public.
- Further, ELSI research needs to be more independent. Far too often those conducting ELSI research are beholden to others. One common concern is that ELSI is often a small portion of a larger basic research grant. In some instances the ELSI component of the grant comes at the unfortunate expense of independent and free expression, particularly when that independent and free expression could run counter to goals of the co-PI's on the grant. ELSI researchers have spoken of the often subtle pressure to come to the right conclusions, toe the line (Morrissey and Walker, 2012). There needs to be more grants that focus primarily on ELSI, or that allow ELSI researchers to be the lead PI's such that they have greater independence in the direction of their research.
- And, ELSI research needs to be more international to mirror the international groups and consortia that conduct today's biomedical research. If ELSI is going to provide useful information and guide researchers and policy makers then it has to better represent the growing international nature of research. The diffusion of ELSI to match the diffusion of the science across borders however, is a non-trivial problem given some of the stark differences in society, morality and the law across the planet. Efforts such as ELSI 2.0 are important pushes towards a more international ELSI community (Kaye et al., 2012).
- With this international nature of science, what may have been the disapproval of a small minority in a particular research direction may grow into a large concern shared by a larger minority or even a majority. However, even when the majority disapproves, science might still need to move forward despite the broad disapproval. This too is a non-trivial decision. ELSI will need to determine red lines for when even basic science research needs to acquiesce to societal pressures.
- Some may argue that ELSI is too big for its own good—that it threatens the funding of other bioethics fields. In all likelihood, ELSI is too narrow in its genomics focus and should be expanding to include other fields such as Neuroscience (Klein, 2010), nanotechnology (Fisher, 2005), or synthetic biology (Calvert and Paul, 2009).

To this end:

I intend this Specialty Section, albeit scientific, academic, and apolitical by design, to include readable content from not just scientists, but lay professionals as well. The Specialty should have broad appeal and be of interest not just as a source to non-scientists with an interest in the science, but also invite those non-scientists to become part of the conversation incentivizing collaborative-cross disciplinary research in not just genomics or even the biosciences, but in all scientific fields that have the potential to raise ethical legal and social concerns. The Specialty Section is intended to use the resources available to speed up the publication process, to provide relevant and readable papers in response to timely issues. The Specialty is intended to be a place of uninhibited sharing of data and information, promoting cross collaborations and conversations. The Specialty Section has an international outlook looking for representation in its editorial board and its authorship of different nationalities and even non-scientific professions that can provide a further heretofore unappreciated understanding of the issues and their repercussions.
